# Update on gut microbiota in cardiovascular diseases

**DOI:** 10.3389/fcimb.2022.1059349

**Published:** 2022-11-10

**Authors:** Buyun Qian, Kaiyu Zhang, Yuan Li, Kangyun Sun

**Affiliations:** Department of Cardiology, The Affiliated Suzhou Hospital of Nanjing Medical University, Suzhou Municipal Hospital, Gusu School, Nanjing Medical University, Jiangsu, Suzhou, China

**Keywords:** gut microbiota, cardiovascular diseases (CVDs), metabolites, association, therapies

## Abstract

In recent years, due to the development and widespread utilization of metagenomic sequencing and metabolomics, the relationship between gut microbiota and human cardiovascular diseases (CVDs) has received extensive attention. A growing number of studies have shown a strong relationship between gut microbiota and CVDs, such as coronary atherosclerosis, hypertension (HTN) and heart failure (HF). It has also been revealed that intestinal flora-related metabolites, such as trimethylamine-N-oxide (TMAO), short-chain fatty acids (SCFA) and bile acids (BAs), are also related to the development, prevention, treatment and prognosis of CVDs. In this review, we presented and summarized the recent findings on the relationship between gut microbiota and CVDs, and concluded several currently known gut microbiota-related metabolites and the occurrence and development of CVDs.

## Introduction

Cardiovascular diseases (CVDs), including coronary atherosclerosis, hypertension (HTN) and heart failure (HF), are important causes of death, resulting in a huge economic and health burden globally (Zoungas et al., 2014; Writing Group et al., 2016). As a chronic disease, in addition to inflammation, diabetes will affect the occurrence and development of CVDs (Haybar et al., 2019). Other factors such as diet and nutritional status also have a more profound effect on CVDs (Brown and Hazen, 2018). Moreover, microbial sequencing analysis has provided a wealth of information about the presence of characteristic gut microbiota associated with CVDs (Jäckel et al., 2017; Kiouptsi and Reinhardt, 2018; Lindskog Jonsson et al., 2018; Haghikia et al., 2022). Therefore, more and more studies have shown that the gut microbiota has a strong link with CVDs.

Human gut is a huge microbial habitat. The adult gut contains hundreds of species of bacteria. Gut flora plays an important role in maintaining body health (Wampach et al., 2017; Wu et al., 2020b; Li and Chen, 2022). Gut microbiota can produce certain biologically active metabolites in the gut. These gut microbiota-related metabolites function in various aspects of host physiology and are known as the ninth system of the human body (O’Hara and Shanahan, 2006; Qin et al., 2010). Intestinal flora can form the intestinal epithelial barrier, regulate immune function, digest nutrients, produce vitamins and prevent the invasion of pathogenic bacteria, which is essential for human health (DeGruttola et al., 2016; Thomas and Jobin, 2020; Wu et al., 2020a; Chen et al., 2021; Cui et al., 2021). When dietary habits, environmental factors, intestinal infections and other factors lead to changes in the human gut microbiome, it can lead to intestinal malnutrition, trigger inflammation and abnormal metabolism and then lead to the occurrence and development of CVDs (Yatsunenko et al., 2012; Naghavi et al., 2015). In this review, we introduced the role of gut microbiota in CVDs and summarized the related metabolites, which may provide new insight into the association between gut microbiota and CVDs (**Table 1**).

**Table 1:** The association between gut microbiota and CVDs.

Types of CVDs	Changes in the gut microbiota	Involvement of gut microbiota metabolites	Mechanism
Coronary atherosclerosis	Increased *Streptococcus*; Increased *Roche*; Increased *Ruminococcus*; Increased *Clostridium*.	TMAO	Cholesterol metabolism ↓; Foam cells ↑.
			Promote the activation of NF-κB; IL-18 ↑; IL-1β ↑.
		BAs	Cholesterol increase ↑; Reduce the risk of atherosclerosis.
		LPS	Foam cells ↑; Cholesterol ↑.
HTN	Increased *Prevotella*; Increased *Bifidobacterium*; Increased *Lactobacillus*.	SCFAs	Knock out of Olfr78 and GPR41, lead to high blood pressure.
		Propionate	Adjust Th17 and lower blood pressure.
HF	Increased *Candida*; Decreased *Faecalibacterium*.	BAs	Regulate the calcium ion concentration.
		SCFAs	Disrupt the intestinal barrier; Promote the translocation of endotoxins into the blood.
		TMAO	Ca^2+^ ↑; Myocardial fibers ↑.
			Induce T-tubule network damage and calcium processing dysfunction.
			Activate NLRP3.

CVDs: Cardiovascular diseases; HTN: Hypertension; HF: Heart failure; TMAO: Trimethylamine-N-oxide; BAs: Bile acids; LPS: Lipopolysaccharide; SCFAs: Short-chain fatty acids.

## Gut microbiota and coronary atherosclerosis

Coronary heart disease (CHD) is based on coronary atherosclerosis and is an important representative of metabolic CVDs. The metagenomic sequencing showed that gut microbiota in patients with atherosclerotic cardiovascular disease differed from healthy individuals, with higher levels of *Streptococcus* and *Enterobacteriaceae* (Ott et al., 2006; Jie et al., 2017). It has been reported that co-abundant gut microbiota and serum metabolites are closely related to CHD severity, and gut microbiota such as *Roseburia*, *Ruminococcaceae* and *Clostridium* may regulate the metabolic activity of bile acids (BAs) and aromatic compounds, which will further affect the progression of coronary atherosclerosis (Liu et al., 2019a). It was revealed that gut microbiota and coronary atherosclerosis are correlated (**Table 1**).

Intestinal dysbiosis can also exert pro-atherosclerotic effects through metabolism-dependent pathways by altering the production of various metabolites, including Trimethylamine-N-oxide (TAMO), BAs, serum indoxylate, protocatechuic acid and lipopolysaccharide (LPS). TMAO is one of the most important metabolites associated with gut microbiota. Studies have shown that the body’s immune system regulation, cholesterol metabolism, oxidative stress and inflammatory responses will all be affected by TMAO to a certain extent, thereby increasing the risk of coronary atherosclerosis. Therefore, increased plasma TMAO concentration can increase the possibility of CVDs (Seldin et al., 2016; Geng et al., 2018; Xie et al., 2021). Wang et al. found that TMAO-dependent upregulation of macrophage scavenger receptor and CD36 expression impaired cholesterol metabolism in macrophages, thereby promoting foam cell production, one of the earliest cellular signals in the progression of coronary atherosclerosis (Wang et al., 2011). Studies have found that elevated levels of TMAO can induce activation of the NF-κB pathway and promote the release of inflammatory cytokines IL-18 and IL-1β, indicating that inflammatory mediators play a role in TMAO-induced endothelial dysfunction (Liu et al., 2019c). Zhu et al. found that TMAO could increase intracellular rectal calcium release from platelets, leading to platelet aggregation and thrombosis (Zhu et al., 2016). This study found that the choline analog 3,3-dimethylbutanol (DMB) has an inhibitory effect on choline TMA lyase activity and can reduce circulating TMAO, thereby attenuating the promoting effect of choline in coronary atherosclerosis (Wang et al., 2015).

TMAO plays an important role in the development of coronary atherosclerosis. Furthermore, coronary atherosclerosis is also affected by cholesterol metabolism, short-chain fatty acids and tryptophan metabolites (Paeslack et al., 2022; Roessler et al., 2022). It has been shown that deconjugated BAs are hydrophobic and can be excreted in feces, which will in turn lower circulating cholesterol and thus reduce the risk of coronary atherosclerosis (Dawson and Karpen, 2015). It was also found that serum indoxylate levels are positively correlated with coronary atherosclerosis and are predictive mechanistic biomarkers of coronary artery disease severity (Hsu et al., 2013). Additionally, the expression of atherosclerosis-related genes is also regulated by protocatechuic acid, including oxidative stress-related AOX1, CYP2E1 or TXNIP, adhesion molecule JAM-A, angiogenesis-related blood vessels endothelial growth factor receptor 2 and so on (Mauray et al., 2012). Moreover, LPS was reported to induce foam cell formation and cholesteryl ester accumulation from native low-density lipoprotein, suggesting that LPS has a pro-atherosclerotic effect (Lakio et al., 2006).

Gut microbiota metabolites are also involved in arterial thrombosis. It was revealed that fecal transplantation of TMAO-rich gut microbiota into germ-free mice can promote platelet function and arterial thrombosis (Huynh, 2020). Nemet et al. found that phenylacetylglutamine can induce platelet hyperresponsiveness through adrenergic receptors (Nemet et al., 2020). Besides, phytoestrogens may also have some prothrombotic or proinflammatory effects (Herrington, 2000).

## Gut microbiota and hypertension

HTN is the most common risk factor associated with CVDs, and as the main risk factor for stroke and CHD morbidity and mortality (Page, 1967), it has always been a hot topic. Recently, studies have shown that the gut microbiota is involved in blood pressure regulation and that abnormal bacterial populations are associated with HTN (Li et al., 2017; Wilck et al., 2017). Compared with healthy individuals, the abundance and diversity of gut microbes in hypertensive patients decreased, and the genus *Prevotella* was significantly increased (Li et al., 2017). In addition, a fecal microbiota transplantation (FMT) study confirmed that the fecal microbiota of patients with HTN can increase the blood pressure in germ-free mice, revealing a close link between gut microbiota and the regulation of blood pressure (Mell et al., 2015). Hence, there exists a link between gut microbiota and HTN (**Table 1**).

In addition to changes in gut microbiota composition, excessive formation of gut microbiota metabolites is also considered to be a key factor in the occurrence of HTN. Bacteria belonging to the genera *Bifidobacterium*, *Lactobacillus*, *Streptococcus* and *Escherichia coli* can produce neurotransmitters within the autonomic nervous system that will alter vascular tone, leading to HTN (Karbach et al., 2016). It was also shown that the higher levels of circulating TMAO are positively associated with the high risk of blood pressure (Ge et al., 2020). Liu et al. found that the use of *Lactobacillus rhamnosus GG* strain can prevent HTN deterioration by reducing the levels of TMAO (Liu et al., 2019b). Besides, short-chain fatty acids (SCFAs) are also of great significance in the regulation of blood pressure and are mainly involved in the regulation of blood pressure mainly through Olfactory receptor 78 (Olfr78) and G protein-coupled receptor 41 (GPR41) (Felizardo et al., 2019). Natarajan et al. found that GPR41 knockout mice exhibited HTN (Natarajan et al., 2016). The activation of GPR41 was found to reduce SCFA-producing bacteria in some patients with hypertensive gut dysregulation (Pluznick, 2014). Meanwhile, Miyamoto et al. showed that Olfr78-deficient mice also exhibited high blood pressure (Miyamoto et al., 2016). Furthermore, studies also have shown that the production of propionate depends on regulatory T cells, and blood pressure can be reduced through regulatory T cells 17 and angiotensin II-induced effectors (Bartolomaeus et al., 2019). Although there exist many studies on the mechanism how gut microbiota influence HTN, the specific mechanism remains unclear and more studies are needed. The regulation of blood pressure by intestinal flora metabolites such as TMAO, SCFA and propionate will provide a new idea for drugs to improve intestinal flora to treat HTN.

## Gut microbiota and heart failure

HF is an irreversible end-stage disease with high mortality, characterized by edema and dyspnea (Ponikowski et al., 2016). The gut hypothesis suggested that reduced cardiac output and increased systemic congestion can cause ischemia and edema of the intestinal mucosa, leading to increased bacterial translocation and increased circulating endotoxin, leading to HF (Krack et al., 2004; Sandek et al., 2007). Studies have found that patients with HF presented the increased levels of pathogenic bacteria such as *Candida* and the decreased levels of anti-inflammatory bacteria such as *Faecalibacterium*, therefore contributing to the development of HF by participating in the regulation of mucosal immune (Pasini et al., 2016). This indicated that there exists a correlation between gut microbiota and HF (**Table 1**).

Gut microbiota metabolites such as SCFAs, TMAO, indoxyl sulfate and LPS also play an important role in the development of HF. Savi et al. demonstrated that TMAO can promote calcium release in healthy mouse cardiomyocytes, thereby altering their contractility (Zabell and Tang, 2017; Savi et al., 2018). The direct dietary TMAO supplementation can lead to higher systemic TMAO levels, increase myocardial fibrosis and induce HF (Organ et al., 2016). It was revealed that TMAO has deleterious effects on adult cardiomyocytes by inducing T-tubule network damage and calcium-handling dysfunction (Jin et al., 2020). TMAO can promote myocardial fibrosis by activating NLRP3 inflammasome-related signaling, suggesting that TMAO may be a potential target for the treatment of HF (Siu et al., 2019). Schuett et al. demonstrated that TMAO increases patient susceptibility to HF by increasing myocardial fibrosis (Schuett et al., 2017). Similarly, Wang et al. found that 3,3-dimethyl-1-butanol (DMB) ameliorated adverse cardiac structural remodeling in overload-induced HF mice by downregulating TMAO levels (Wang et al., 2020). Indoxyl sulfate exacerbates cardiac fibrosis, cardiomyocyte hypertrophy, and atrial fibrillation (Yisireyili et al., 2013; Aoki et al., 2015). Besides, Mayerhofer et al. found that BAs can play a role in the cardiovascular function by reducing heart rate by modulating channel conductance and calcium dynamics in atrial and ventricular cardiomyocytes, as well as modulating vascular tone (Mayerhofer et al., 2017). SCFAs have gut protective effects. Decreased SCFAs will lead to disruption of the intestinal barrier, facilitating translocation of endotoxins into the blood circulation, ultimately leading to HF (Tang et al., 2012; Nagatomo and Tang, 2015). SCFAs can also promote cardiac repair after HF by inducing CX3CR1+ cells (Tang et al., 2019). Furthermore, LPS can damage the mucosal barrier function of the intestine, increase intestinal permeability, thereby increasing inflammatory cytokines, which is closely related to the occurrence of HF (Lam et al., 2012b).

## Microorganism-targeted therapies

There existed several microorganism-targeted therapies used in CVDs (**Table 2**). FMT, which refers to the replacement of enteric pathogens by introducing the fecal contents of healthy subjects into the gastrointestinal tract of patients, is an effective method to directly introduce the gut microbiota (Colman and Rubin, 2014; Zhang et al., 2019). Studies have shown that FMT can eliminate the increased Bacteroides/Firmicutes ratio and reduce inflammation in cardiomyocytes, thereby reducing myocarditis in mice (Kim et al., 2018). In addition, clinical studies have shown that FMT can quickly restore the gut microbiota of healthy people after the use of antibiotics (Taur et al., 2018).However, the use of FMT is currently limited due to the transfer of endotoxins or infectious agents that may lead to new gastrointestinal complications (Brandt, 2013; De Leon et al., 2013).

**Table 2:** Microorganism-targeted therapies.

Types of CVDs	Treatment methods	Authors	Role
Myocarditis	FMT	Kim et al. (Kim et al., 2018)	Reduce inflammation and myocarditis.
–	FMT	Taur et al. (Taur et al., 2018)	Restore the healthy gut microbiota.
–	Fiber-rich diet	Foye et al. (Foye et al., 2012)	Promote the growth of beneficial symbiotic bacteria and inhibit the growth of opportunistic pathogens.
–	Dietary Intervention	Xiao et al. (Xiao et al., 2014)	Reduce *Enterobacteriaceae* pathogenic bacteria and increase intestinal protective bacteria.
HTN	High-fiber diet	Marques et al. (Marques et al., 2017)	Lower blood pressure.
HTN	*Bifidobacterium breve* and *Lactobacillus fermentum*	Chi et al. (Chi et al., 2020)	Lower blood pressure.
Myocardial infarction	*Lactobacillus plantarum*	Lam et al. (Lam et al., 2012a)	Reduce myocardial infarction size.
Myocardial infarction	*Lactobacillus rhamnosus* GR-1	Gan et al. (Gan et al., 2014)	Reduce myocardial infarction size.
HF	*Saccharomyces boulardii*	Coatanza et al. (Costanza et al., 2015)	Have therapeutic effect on patients with HF.
HF	Antibiotics	Zhou et al. (Zhou et al., 2018)	Reduce damage to cardiomyocytes.
–	Rifaximin	Ponziani et al. (Ponziani et al., 2017)	Have anti-inflammatory effects and modulate gut microbiota.
HF	Polymyxin B and tobramycin	Conraads et al. (Conraads et al., 2004)	Reduce the level of inflammatory factors in the gut of patients with HF.
HF	DMB	Chen et al. (Chen et al., 2017)	Reduce ventricular remodeling.
Atherosclerosis	Resveratrol	Chen et al. (Chen et al., 2016)	Alleviate TMAO-induced atherosclerosis.
Myocardial infarction	Exercise	Liu et al. (Liu et al., 2017)	Prevent myocardial infarction.
Atherosclerosis	Curcumin	Ghosh et al. (Ghosh et al., 2014)	Attenuate atherosclerosis.

FMT, Fecal microbiota transplantation; DMB, 3,3-dimethylbutanol; HTN, Hypertension; HF, Heart failure; TMAO, Trimethylamine-N-oxide.

Dietary intervention to regulate the treatment of CVDs has broad prospects (Appel et al., 1997; Estruch et al., 2013). Studies have shown fiber-rich diet can promote the growth of beneficial symbiotic bacteria and inhibit the growth of opportunistic pathogens (Foye et al., 2012). Xiao et al. found that dietary intervention with whole grains and traditional Chinese medicine foods can reduce *Enterobacteriaceae* pathogenic bacteria and increase intestinal protective bacteria such as *Bifidobacterium* (Xiao et al., 2014). In addition, a high-fiber diet can increase the acetic acid-producing microbiota, which in turn lowers blood pressure (Marques et al., 2017).

Among the numerous bacteria in the host gut, some are beneficial, and additional enhancement of these bacteria may lead to positive outcomes, resulting in the use of probiotics (Ojetti et al., 2009). It was found that the probiotics *Bifidobacterium breve* and *Lactobacillus fermentum* can have antihypertensive effects by restoring gut microbiota balance and preventing endothelial dysfunction (Chi et al., 2020). Lam et al. found that *Lactobacillus plantarum* could improve ventricular function and reduce myocardial infarction size (Lam et al., 2012a). In addition, similar results were obtained by treating myocardial ischemia rats with *Lactobacillus rhamnosus* GR-1 (Gan et al., 2014). *Saccharomyces boulardii* can also reduce the level of inflammatory markers and serum creatinine, which has therapeutic effect on patients with HF (Costanza et al., 2015). Although probiotics are safe, there is a lack of supervision, which may increase the risk of probiotics transferring to blood and lead to sepsis (Kochan et al., 2011).

Antibiotics affect the structure of the gut microbiota, which in turn treats CVDs. Study shows that antibiotic injections can eliminate shifts in gut microbiota, reduce damage to cardiomyocytes (Zhou et al., 2018). Several studies have found that antibiotics can reduce inflammation, such as rifaximin, which has anti-inflammatory effects and modulates gut microbiota (Ponziani et al., 2017), and polymyxin B and tobramycin can reduce the level of inflammatory factors in the gut of patients with HF (Conraads et al., 2004), which has important implications for the treatment of CVDs.

DMB treatment can reduce TMA production, limit the conversion of TMA to TMAO, and reduce ventricular remodeling (Chen et al., 2017). Furthermore, resveratrol from Polygonum cuspidatum can alleviate TMAO-induced atherosclerosis by remodeling the microbiota and reducing TMAO levels (Chen et al., 2016).

Besides, exercise can boost *Firmicutes* to *Bacteroides* ratio (Petriz et al., 2014; Lambert et al., 2015), increase levels of bacterial metabolites (Allen et al., 2018) and prevent myocardial infarction (Liu et al., 2017). But the effects of exercise on the gut microbiome are transient and reversible (Lira et al., 2010).

There are also other treatments, which can be used for CVDs. Ghosh et al. found that curcumin attenuates atherosclerosis by modulating intestinal barrier function (Ghosh et al., 2014). Additionally, berberine, derived from the Chinese herb Coptis chinensis, can modulate the gut microbiota, which in turn affects CVDs (Wang et al., 2019). In summary, Microorganism-targeted therapy mainly regulates CVDs through FMT, dietary interventions, and probiotics.

## Conclusions and prospects

**Figure 1:**
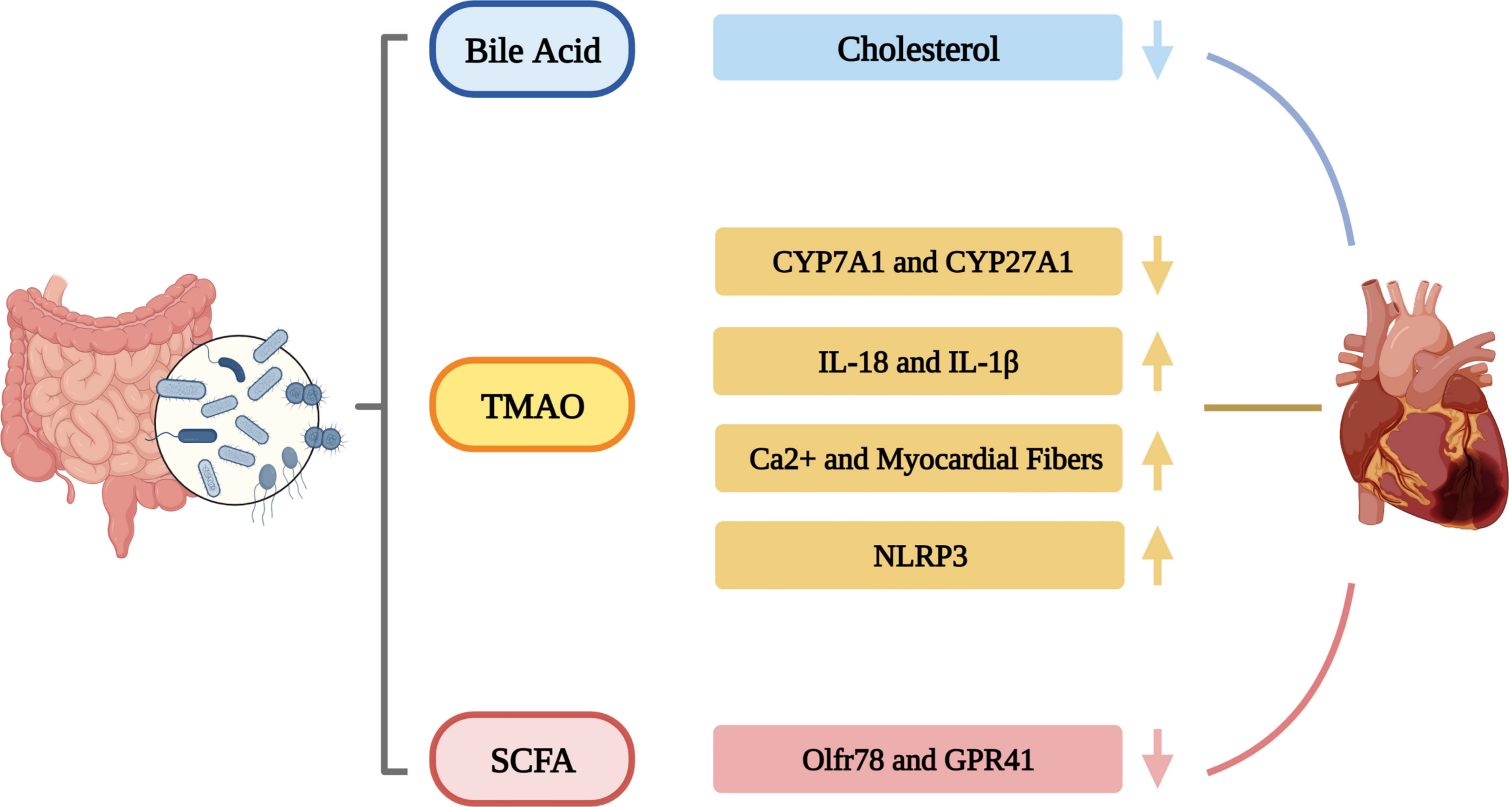
The roles of gut microbiota in CVDs.

A large number of research results show that the gut microbiota is involved in the occurrence and development of CHD, HTN and HF, and plays an important role in it. The gut microbiota influence CVDs through immune regulation, the inflammatory response, gut barrier integrity, metabolic homeostasis. CVDs, in turn, also affect the structure and function of the gut microbiota (**Figure 1**). In addition to traditional culture methods, more advanced technologies such as metagenomics and metabolomics have become important means to study the human gut microbiota. Numerous studies have observed the relationship between gut microbiota and CVDs and proposed various potential mechanisms of action, especially mechanisms such as metabolic pathways. A better understanding of the human gut microbiota can provide more potential treatments for patients with CVDs on the basis of current clinical traditional drug treatments, such as dietary adjustments, rational use of probiotics and antibiotics, and even FMT, etc., bringing more possibilities for the prevention and treatment of CVDs. At present, most studies on the correlation between gut microbiota and CVDs are based on animal experiments and the mechanism between gut microbiota and CVDs is still not fully understood. Although TMAO is a potential biomarker for CVDs development, other gut microbiota or related metabolites should be explored as early CVDs markers. In addition, exploring the immune mechanisms of CVDs also helps us analyze how gut microbiota metabolites interfere with disease at the molecular level. More experiments are needed to explore the link between gut microbiota and CVDs, and further clinical studies are needed. Some approaches based on gut microbiota for the treatment of CVDs are still in clinical trials and have potential advantages as well as limitations. Therapeutic strategies to improve the gut microbiota are potential avenues for the treatment of CVDs.

## Author contributions

BQ had the idea for the article. KZ performed the literature search and data analysis. YL and KS drafted and critically revised the work. All authors contributed to the article and approved the submitted version.

## Funding

This study was supported by Nanjing Medical University Gusu College Scientific Research Fund (NO. GSKY20210202).

## Conflict of interest

The authors declare that the research was conducted in the absence of any commercial or financial relationships that could be construed as a potential conflict of interest.

## Publisher’s note

All claims expressed in this article are solely those of the authors and do not necessarily represent those of their affiliated organizations, or those of the publisher, the editors and the reviewers. Any product that may be evaluated in this article, or claim that may be made by its manufacturer, is not guaranteed or endorsed by the publisher.

## Abbreviations

CVDs, Cardiovascular diseases; TMAO, Trimethylamine-N-oxide; SCFAs, Short-chain fatty acids; BAs, Bile acids; CHD, Coronary atherosclerotic heart disease; DMB, 3,3-dimethylbutanol; LPS, Lipopolysaccharide; HTN, Hypertension; FMT, Fecal microbiota transplantation; HF, Heart failure
